# The Effect of Roasting on the Protein Profile and Antiradical Capacity of Flaxseed Meal

**DOI:** 10.3390/foods9101383

**Published:** 2020-09-30

**Authors:** Katarzyna Waszkowiak, Beata Mikołajczak

**Affiliations:** 1 Department of Gastronomy Science and Functional Foods, Faculty of Food Science and Nutrition, Poznan University of Life Sciences, Wojska Polskiego 31, 60-624 Poznań, Poland; 2 Department of Meat Technology, Poznan University of Life Sciences, Wojska Polskiego 31, 60-624 Poznań, Poland; beata.mikolajczak@up.poznan.pl

**Keywords:** flaxseed, defatted meal, roasting, SDS-PAGE protein profile, antioxidative capacity, Maillard reaction product

## Abstract

Roasting is more and more often used as a pre-treatment of flaxseeds. However, the process can influence flaxseed proteins that may be crucial for their properties. The aim of this research was to study changes in the electrophoretic protein profile (SDS-PAGE) and the antiradical capacity of flaxseed meals after roasting. The roasting temperature (160, 180, and 200 °C) and flaxseed cultivars (golden and brown seed) were factors including in the study. The free (F-MRP) and bound-to-protein (B-MRP) Maillard reaction products were also analyzed. The most significant changes in the SDS-PAGE protein profiles of roasted seeds of each of the tested flax cultivars were observed for the 13 kDa protein fraction (decrease) and for the 19 kDa and 17 kDa fractions (increase). The research revealed a significant correlation between the roasting temperature and B-MRP content, and changes in the percentage share of those three protein fractions. The antiradical capacity of roasted flaxseeds decreased, as compared with untreated seeds. After roasting at 200 °C the antiradical capacity of flaxseeds improved slightly, probably due to the MRP formation, but it was still significantly lower than that of the raw seeds. The research provides novel information about key protein fractions that seem to be important changing during heat treatment.

## 1. Introduction

Since ancient times, flaxseeds (*Linum usitatissimum* L.) have been included in the diet of humans and animals. The health benefits of flaxseeds are mostly attributed to the content of compounds such as α-linolenic fatty acid, lignan secoisolariciresinol, and insoluble and soluble dietary fiber [1]. However, flaxseeds and flaxseed cakes are also a valuable source of protein [2,3]. The crude protein content (N × 6.5) amounts to about 35–45% of the dry oil-free mass of flaxseeds [2]. The amino acid profile, digestibility, and biological value of flaxseed proteins have been reported to be comparable to soy proteins [4]. Flaxseed proteins are usually divided into high and low molecular weight (MW) fractions [5]. The major high MW fraction of flaxseed protein, often termed as linin (its MW estimated with various analytical methods is 252–320 kDa), amounts to about 58–66% of total flaxseed proteins [6,7]. Linin has 11–12 S globular structure consisting of five unidentical polypeptide subunits with MWs of 14.4 kDa, 24.6 kDa, 30.0 kDa, 35.3 kDa, and 50.9 kDa (SDS-PAGE MW distribution) [7]. The major low MW component of flaxseed protein is called conlinin. It is a 2S albumin-type structure consisting of a single polypeptide chain (15–17 kDa), which makes 20–42% of the total flaxseed proteins [2,6]. The 2S proteins are water-soluble (about 93%) due to their small size [8]. Other low MW structural and physiological proteins (e.g., oleosin, hirudin) were also found in flaxseeds [2].

Flaxseed protein is an interesting source of unique peptides and proteins. Some of them are endogenous, e.g., 0.9–1.1 kDa cyclolinopeptides [9], 7 kDa lunasin-like peptide [10], and 25 kDa linusitin [11], while others are products of hydrolysis [12]. They exhibit anti-inflammatory, hypotensive, immunosuppressive, and anti-fungal properties [12,13,14]. Flaxseed proteins and peptides also exhibit various functional properties, e.g., water and fat absorption, emulsification, and foaming capacity [3,15], which are important in the food industry. These properties of flaxseed proteins recently encourage researchers to focuses on methods of efficient extraction and isolation [16,17]. The influence of various processes on modification and improvement of functional properties of flax proteins was also investigated. Yu et al. [18] considered atmospheric pressure plasma jet as an effective strategy to improve the functionality (foaming and emulsifying properties) and antioxidant activities of flaxseed protein. Juodeikiene et al. [19] used high-frequency ultrasound for the flaxseed protein functionalization, and they found that functional properties of proteins (emulsifying, foaming, and gelling) significantly depended on the medium pH and ultrasound intensity. The properties of flaxseed proteins also cause more and more frequent attempts to use them in food production (e.g., bread, gluten-free products, and meat products) [20,21,22].

Roasting is more and more often used as a pre-treatment of flaxseeds when they are to be used as a food ingredient [23] because it improves their flavour. It also significantly reduces the cyanogenic glycoside content in flaxseeds [24]. The process alters the cellular structures of seeds and increases their capacity to be broken up by chewing [25]. Moreover, heating before cold-pressing of oilseeds generally increases oils yield [26]. On the other hand, roasting damages the cell structure [27] and increases the sensitivity of valuable flaxseed compounds to changes induced by thermal treatment [28,29,30,31]. An earlier study by Waszkowiak, Siger et al. [28] showed that the roasting of flaxseeds at a temperature of 160–220 °C considerably influenced the nutritional quality of flaxseed oil (e.g., the content of total tocopherol decreased, but the content of plastochromanol-8 increased) and deteriorated its oxidative stability. Other studies [29,30] revealed changes in the percentage share of individual protein fractions in the protein profiles of roasted (in the ranges of 160–200 °C and 8–24 min) and steamed (100 °C, 16 min) flaxseeds. However, the differences between the cultivars were not assessed in these studies. Changes in the protein fractions may be caused by both protein structural changes and their interactions with other seed compounds (e.g., as a result of the formation of Maillard reaction products). It may be crucial for the nutritional value and functional properties of flaxseed proteins. So far, there has been no comprehensive study showing the effect of both roasting temperature and cultivar on changes in the protein profile as well as the antioxidative capacity of flaxseed meals.

The aim of the research was to study changes in the electrophoretic protein profile (SDS-PAGE) and antiradical capacity (oxygen radical absorbance capacity assay with fluorescein as a fluorescent probe–ORAC_FL assay) of flaxseed meals after roasting. The roasting temperature (160, 180, and 200 °C) and flaxseed cultivars (golden and brown seed) were factors including in the study. The free (F-MRP) and bound-to-protein (B-MRP) Maillard reaction products were also analyzed.

## 2. Materials and Methods

### 2.1. Materials

The research materials were flaxseeds (*Linum usitatissimum* L.) of three cultivars: golden seed Jantarol, Oliwin, and brown seed Szafir (IHAR, Borowo, Poland; year of production: 2017). The seeds were stored in jute bags at room temperature. The moisture, protein and fat contents of raw seeds (Table S1) were 69.7 ± 0.3, 220.9 ± 8.7 and 386.4 ± 0.7 g/kg for Szafir cultivar; 58.2 ± 8.7, 213.7 ± 0.7, and 400.1 ± 1.1 g/kg for Oliwin; and 54.2 ± 1.3, 170.4 ± 3.7, and 421.7 ± 6.0 g/kg for Jantarol, respectively. The contents of soluble and insoluble dietary fibre (determined according to the Association of Official Analytical Chemists method–AOAC 991.43 [32]) were 209.4 ± 10.5 and 71.3 ± 10.5 g/kg for Szafir, 212.8 ± 11.3 and 74.7 ± 4.6 g/kg for Oliwin, and 221.0 ± 1.8 and 93.6 ± 11.6 g/kg for Jantarol, respectively.

The seeds were roasted in the oven (convection oven, CCC series, Rational, Landsberg am Lech, Germany) for 8 min at temperatures of 160, 180, and 200 °C. The thermal treatment of the flaxseeds was conducted according to the procedure described in detail by Waszkowiak et al. [28]. The parameters were selected on the basis of the previous results [25,29,30,33] in order to maintain the acceptable sensory quality of roasted flax seeds. The seeds were roasted in a thin layer (3 mm) to achieve homogenous thermal stress.

Defatted flaxseed meals from the raw and roasted test samples were used for analyses of the electrophoretic protein profile, antiradical capacity and MRP formation. The raw and roasted flaxseed samples were ground in a ZM 200 mill (1 mm sieve, Retsch, Haan, Germany). Double cold oil extraction with n-hexane (ground flaxseed to solvent ratio of 1:3 *w*/*v*) was performed [34,35]. Then the meals were ground in a colloidal mill (Foss, Hilleroed, Denmark) to standardize the composition of the tested material. The meals were cold-stored at 4 °C.

### 2.2. Standards and Reagents

Trichloroacetic acid (TCA), 6-hydroxy-2,5,7,8-tetramethylchromane-2-carboxylic acid (Trolox) and 2,2′-azobis (2-amidinopropane) dihydrochloride (AAPH) were purchased from Sigma-Aldrich (Munich, Germany). Bromophenol blue sodium salt (3′,3″,5′,5″-tetrabromophenol sulfophthalein sodium salt), 2-mercaptoethanol, acrylamide, N,N′-methylenebisacrylamide, sodium dodecylsulphate (SDS), and Coomassie Brilliant Blue R-250 were purchased from SERVA Electrophoresis GmbH (Munich, Germany). PageRuler Plus Protein Ladder 10 to 250 kDa was purchased from Thermo Fisher Scientific (Waltham, MA, USA), and 2-D Quant Kit from GE Healthcare Bio-Sciences (Marlborough, MA, USA). Fluorescein sodium salt was purchased from Fluka (Everett, WA, USA). Pronase E (4,000,000 PU/g) was purchased from Merck (Darmstadt, Germany). Other solvents and reagents of analytical (ACS) or HPLC grade were purchased from Sigma-Aldrich, SERVA Electrophoresis (Munich, Germany) or POCH (Gliwice, Poland).

### 2.3. Chemical Composition Analysis

The chemical composition of the untreated (raw) and roasted flaxseed samples was determined according to ISO standards. The moisture content [36], protein content (the Kjeldahl method, with a Kjeltec 2200 distillation unit, Foss, Hilleroed, Denmark; [37] and fat content (the extraction-weight method with the Soxtec HT6 system, Foss; [38] were all analyzed (see Supplementary Materials Table S1).

### 2.4. Electrophoretic Protein Separation (SDS-PAGE)

**Figure 1:**
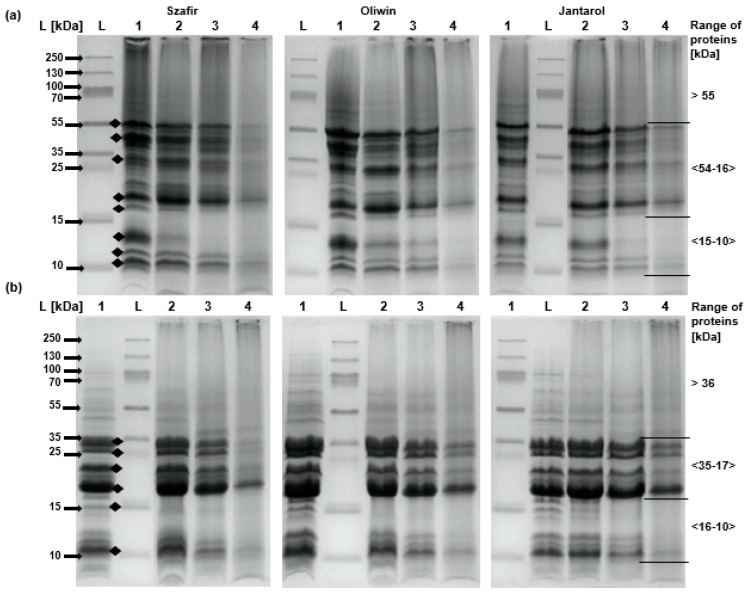
Analysis of untreated and thermal pretreated flaxseed samples by SDS-PAGE electrophoresis in non-reducing (**a**) and reducing (**b**) conditions. Line L—protein ladder (nine proteins spanning: 10, 15, 25, 35, 55, 70, 100, 130, and 250 kDa); 1—untreated; 2—roasted 160 °C, 8 min; 3—roasted 180 °C, 8 min; 4—roasted 200 °C, 8 min. Black dots indicate major bands of the protein profile.

The electrophoretic analysis (SDS-PAGE) of defatted meals from untreated and roasted flaxseeds was performed under reducing (R) and non-reducing (NR) conditions (i.e., with or without DTT and 2-mercaptoethanol in a sample buffer and reservoir buffer for electrophoresis, respectively) according to the procedure described by Waszkowiak et al. [29]. The separation was performed in 15% polyacrylamide separating gel. The protein content in each separated sample was 12 μg (it was measured with a 2-D Quant Kit, GE Healthcare Bio-Sciences, Marlborough, MA, USA). Gel images were acquired with an ImageMaster VDS imaging system and analyzed with the ImageMaster 1D Elite v.4.0 programme (Pharmacia Biotech, Vienna, Austria). Semi-quantitative analysis of selected peaks with a PageRuler Plus Protein Ladder 10 to 250 kDa (Thermo Scientific, Waltham, MA, USA) was based on the molecular weight (MW) expressed as kiloDaltons (kDa). Computations were based on the assumption that the area of a single protein band amounted to the percentage ratio of the area of all separated protein bands (Figure 1), which was 100%. The percentage share of protein bands was analyzed statistically.

### 2.5. Maillard Reaction Product Analysis (Browning Index)

The Maillard reaction product (MRP) was measured with the method described by Palombo et al. [39] with some modifications [40]. Bound-to-protein and free forms of the MRP were determined.

For determine of the total MRP (both bound-to-protein and free forms), digestion (40 °C for 48 h) of a sample with Pronase E was applied to release MRP compounds from protein binding, followed by deproteinisation with TCA (the final concentration of 7%) and centrifugation at 15,000× *g* for 15 min at 4 °C. For analysis of free MRP (F-MRP), a sample was only deproteinised with TCA and centrifuged. The detailed procedure was described by Waszkowiak et al. [28].

In the case of both free and total MRP, the absorbance of supernatants was measured (AU420) at a wavelength of 420 nm (Specord 40 spectrophotometer, Analytik Jena, Jena, Germany). The absorbance at 550 nm (AU550) was also measured to allow for any turbidity in the sample [40]. The browning indexes (BI) for free and total MRP were based on the following equation: BI (AU_420*_) = AU420–AU550 and expressed as corrected arbitral absorbance unit (AU_420*_) per g of sample. The bound MPR (B-MRP) was calculated by subtracting the BI of free MRP from the BI of total MPR.

### 2.6. Antiradical Capacity—ORAC_FL Assay

The oxygen radical absorbance capacity assay with fluorescein as a fluorescent probe (ORAC_FL) assay by Ou et al. [41], which measures scavenging activity against peroxyl radical induced by AAPH, were carried out following the previously described procedure [42]. The assay was conducted in 75 mM phosphate buffer (pH 7.4) at 37 °C. The fluorescence of fluorescein was recorded at fluorescence spectrophotometer (Hitachi F-2700, Tokyo, Japan; 493 nm excitation and 515 nm emission wavelength) before and every 5 min after APPH addition for 40 min.

Samples (0.025 g of the defatted meals) were extracted 1 h with 25 mL of deionised water at room temperature by periodical mixing in a vortex. Next, they were centrifuged at 15,000× *g* for 15 min at 4 °C (Heraeus Megafuge 40R centrifuge, Thermo Fisher Scientific, Santa Clara, CA, USA). Supernatants were collected and filtered through Whatman^®^ Grade 1 filter paper. Then the supernatants were diluted to concentrations within the assay activity ranges (the final concentrations were: 0.25 g/L—untreated seeds and 0.50 g/L—roasted seeds). Trolox solutions (0–100 μmol/L) were used as standards. The results were calculated by subtracting the areas under the fluorescein decay curves for the control sample and the test sample (net area) and were expressed as μmol of Trolox equivalents (TE) per gram of defatted meal. Three independent analyses were performed for each sample.

### 2.7. Statistical Analyses

The roasting was conducted three times, with an individual experiment for each cultivar. All analyses were performed in triplicate. The results were shown as means ± standard deviation (SD).

The results were analyzed statistically with the Statistica v.13.1 software (StatSoft, Tulsa, OK, USA). The effects of the flaxseed treatment (L = 4: untreated and roasted for 8 min. at 160 °C, 180 °C, 200 °C) and cultivar (L = 3: Oliwin, Jantarol, and Szafir) were analyzed. Analysis of variance (ANOVA) for a completely randomised design (CRD) was carried out. Then, post hoc Tukey’s test was applied at a significance level *p* < 0.05 to compare the means and show the effect of the pre-treatment.

Relationships between the variables were examined with linear regression analyses, and Pearson’s correlation coefficients (*r*) were calculated. Principal component analysis (PCA) was applied to the datasets from the treatment conditions and various flaxseed cultivars using the mean values of six variables, i.e., F-MRP; B-MRP; ORAC_FL; and 17-kDa, 13-kDa and 19-kDa shear in SDS-PAGE protein profiles.

## 3. Results and Discussion

### 3.1. Effect of Flaxseed Roasting on Protein Profile

The chemical composition of the untreated (raw) and roasted flaxseed samples are shown in the Supplementary Materials (Table S1).

The electrophoretic protein separations (SDS-PAGE NR and SDS-PAGE R) of defatted flaxseed meals and the percentage share of the main protein fractions in the protein profiles are reported in Figure 1 and Table 1 and Table 2, respectively.

The SDS-PAGE NR meal profiles of all the flaxseed cultivars consisted of 13 components. Eight main protein bands (with MW of ≈ 53–11 kDa) were marked (black dots in Figure 1a) and described as 1a–8a (Table 1). The SDS-PAGE R profiles (Figure 1b) showed six main protein bands with MW of ≈32–11 kDa, which were described as 1b–6b (Table 2). These results were similar to the findings of our previous studies [29,30]. The protein profile of the untreated flaxseeds also corresponds with the results of SDS-PAGE profiles described previously for flaxseed meal [17] and flaxseed cake [43].

For selected cultivars (Oliwin, Jantarol, and Szafir), the SDS-PAGE profiles of the untreated samples showed similar pictures with a slight exception. The SDS-PAGE NR profile of Jantarol was characterized by the lower intensity of the 13 kDa protein band than the Oliwin and Szafir profiles. This protein fraction in the seeds of the Jantarol, Szafir, and Oliwin cultivars amounted to 19%, 25.9%, and 28.5%, respectively (Table 1).

The roasting changed the share of the main fractions in the protein profiles (Figure 1). The most significant changes in the SDS-PAGE NR profiles of the roasted flax seeds of each cultivar (Figure 1a and Table 1) were observed in the protein fractions at MWs of ≈13 kDa (6a) and ≈19 kDa (4a), and these changes were related to the roasting temperature (r = −0.914, *p <* 0.0001 and r = 0.781, *p* = 0.003, respectively). The share of 13 kDa proteins decreased considerably after roasting (Table 1). As the roasting temperature increased, the greatest changes in the percentage share of the 13 kDa fraction were observed in the brown seed Szafir cultivar. The share of this fraction in the protein profiles of the untreated sample and those roasted at 160 °C and 180 °C amounted to 25.9%, 4.6% and <1%, respectively. The band disappeared from the protein profile of the Szafir seeds roasted at 200 °C (Figure 1a). As far as the golden-seed Oliwin and Jantarol cultivars are concerned, the share of the 13 kDa protein fraction in the protein profiles of the untreated samples amounted to 28.5% and 19%, in the samples roasted at 160 °C—10.3% and 16.9%, and in those roasted at 180 °C—3.7% and 2.7%, respectively. As for the Szafir seeds, the fraction disappeared from the protein profile of the Oliwin and Jantarol samples roasted at 200 °C. On the other hand, the percentage of the 19 kDa fraction in the protein profiles of the roasted seeds was significantly higher than in the untreated ones (Table 1). The share of this fraction amounted to 12.8–17.3% in the untreated samples, 18.9–25.2% in the samples roasted at 160 °C, 26.7–29.5% in those roasted at 180 °C and 41–46.8% in those roasted at 200 °C. It is noteworthy that these changes seem to correspond with the changes in the 13 kDa fraction.

As far as the SDS-PAGE R protein profiles are concerned (Figure 1b), in comparison with the untreated seeds the intensity of the protein bands in the seeds roasted at 180 °C and 200 °C decreased significantly at MW ≤ 15 kDa, irrespective of the flax cultivar. Moreover, there were significant changes in the protein profiles of roasted seeds of all cultivars at an MW of ≈17 kDa (4b; Table 2), which were also related to the roasting temperature (r = 0.694, *p* = 0.012). The protein fraction in the untreated samples of the Szafir, Oliwin, and Jantarol seeds amounted to 20.4%, 22.1%, and 23.1%, respectively. The share of the 17 kDa fraction in the protein profile of the samples roasted at 200 °C increased to 63.8%, 52.2%, and 49.5%, respectively. At MWs of 20 kDa and 15 kDa, the protein fractions disappeared from the profiles of the samples roasted at 200 °C (except the Oliwin seeds at 20 kDa).

The changes in the SDS-PAGE protein profiles of roasted flaxseeds may have been caused by structural modifications of their proteins such as denaturation, aggregation, degradation, and cross-linking with polypeptides and other compounds [44]. Moreover, these changes may also have been induced by oxidation and Maillard reaction. The study by Yu [45] showed that the roasting of flaxseeds at 165 °C significantly changed the secondary structure of proteins, inducing an increase in the β-sheet structure to the α-helix structure ratio. It was attributed to the aggregation of proteins and the formation of an intramolecular β-sheet structure during the thermal treatment. Yu [45] also found that proteins and other compounds of the flaxseed matrix formed complexes because protein solubility decreased but the content of proteins bound to cell walls and bound to lignin, and/or Maillard reaction proteins increased. On the other hand, our previous research [28] on the effect of roasting (at temperatures of 160–220 °C) on flaxseed oil quality showed that oil oxidation stability deteriorated significantly. Oil oxidation in situ was particularly significantly accelerated by roasting at ≥200 °C. The acceleration of the oxidation process may also have influenced structural modifications of flaxseed proteins, which resulted in changes in the protein profiles of the flaxseeds roasted at 200 °C.

It is noteworthy that the protein fraction in the roasted flaxseeds decreased at an MW of 13 kDa but increased at MWs of 19 kDa (SDS-PAGE NR) and 17 kDa (SDS-PAGE R) in a treatment-dependent manner. These changes in proteins were also observed in our previous study after roasting flaxseeds for 8, 16, and 24 min at 160 °C [30]. They may have been related to protein degradation, aggregation or formation of complexes with other seed compounds. Changes in the protein fraction of 13 kDa may be characteristic of thermally treated flaxseeds because a similar decrease in this fraction was also observed in steamed flaxseeds [29,30]. The results of studies by Liu et al. [46] and Tehrani et al. [13] suggest that the 13 kDa proteins, which are probably water-soluble albumin-like proteins, could be crucial for the functional and biological properties of flaxseeds. Liu et al. [46] showed that proteins at a similar MW were important for the emulsification ability of flaxseed extracts. Tehrani et al. [13] reported that they showed an antibacterial capacity. Further research is necessary to find whether roasting affects these properties.

### 3.2. Effect of Flaxseed Roasting on the Formation of Free and Bound-To-Protein Maillard Reaction Product (MRP)

**Figure 2:**
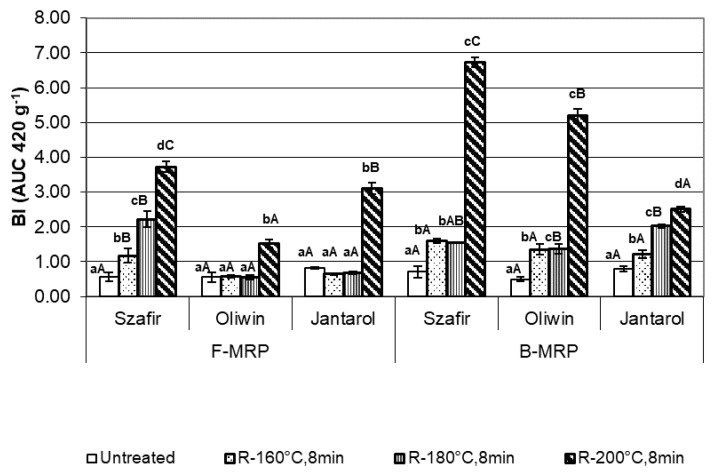
Free (F-MPR) and bound-to-protein (B-MPR) Maillard reaction products of the untreated and roasted flaxseeds, expressed as Browning Index (BI, AU420*/g flaxseed meal). Bars (means ± SD) marked with different letters are significantly different (one-way ANOVA, *p* < 0.05, and post hoc Tukey’s test): a, b, c, d—pre-treatment effect, A, B, C—cultivar effect.

The analysis of the MRP formation (based on BI values, Figure 2) showed that both free (F-MRP) and bound-to-protein (B-MRP) forms of Maillard reaction products were formed during the roasting of flaxseeds, regardless of the cultivar.

The content of F-MRP increased gradually with the roasting temperature of the brown Szafir seeds. It was 6.5 times greater in the seeds roasted at 200 °C than in the untreated seeds. The F-MRP values of the golden seed Oliwin and Jantarol were significantly greater only after roasting at 200 °C.

The B-MRP content in the seeds of all tested cultivars roasted at 160 °C and 180 °C was about 1.5–2.7 times greater (statistically significant difference) than in the untreated seeds. Similar to F-MRP, the most dynamic increase in B-MRP was found after the roasting of flaxseeds at 200 °C. The B-MRP content in the Szafir and Oliwin seeds was significantly greater than in the Jantarol seeds (the values were about 10 and three times greater than in the untreated seeds, respectively).

There was a significant relationship between the roasting temperature and B-MRP content (r = 0.584, *p* = 0.046), but the relationship between the roasting temperature and F-MRP (r = 0.497, *p* = 0.100) was statistically insignificant.

The results showed that during the roasting of flaxseeds Maillard reaction products may be formed. It is possible that melanoidins were formed, i.e., brown nitrogen-containing Maillard reaction products. When Maillard reactions take place at a relatively high temperature (above 150 °C), in a short time and at low moisture during seed roasting [47], (like in our study), oligo- or polysaccharides in a matrix can react as complete molecules at reducing end and additional side chains could be formed by transglycosylation. As a result, high MW melanoidins with a carbohydrate skeleton may be formed. Alternatively, high MW melanoidins with a protein skeleton may be produced by the reaction of sugar or carbohydrate degradation products with proteins. Low MW melanoidins may also be formed under these conditions when sugar or carbohydrate degradation products react with amino acids and then they may be used to produce high MW melanoidins [47]. Low content of free reducing sugars [48] may limit MRP formation in roasted flaxseeds. The study by Epaminondas et al. (2011) showed that the degradation of triglycerides and cell wall carbohydrates may occur during the heating of flaxseeds above 170 °C. Therefore, it could be assumed that the latter way of melanoidin formation is crucial for roasted flaxseeds and these degradation products interact with some flaxseed proteins and form high MW Maillard reaction products. Such products were probably identified in our study as B-MRP (Figure 2). This assumption is in agreement with our findings concerning the changes in the protein profile of the flaxseeds after roasting above 160 °C, especially at 200 °C. The decrease in the band intensity (Figure 1) and percentage (Table 1 and Table 2) of some proteins in both the SDS-PAGE NR and R profiles of the roasted flaxseeds, especially those at MW ≤ 15 kDa, may indicate their contribution to the formation of high MW melanoidins. The contribution of thermally labile 13 kDa protein may be important for the process. It may also explain the lower intensity of bound-to-protein MRP formation in the Jantarol seeds after roasting at 200 °C, as compared with the Szafir and Oliwin samples, because the raw Jantarol seeds were characterized by lower percentage of 13 kDa in their protein profile. However, further research is necessary to confirm these suppositions.

During the roasting of flaxseeds at high temperatures, the formation of melanoidin molecules intensified. This phenomenon can be explained by the processes occurring at a temperature higher than 170 °C, which was described by Epaminondas et al. [49]. It seems that their intensification during roasting at high temperature may also favor MRP formation. Bekedam et al. [50] found that more intensive (darker) roasting of coffee also led to the formation of high MW melanoidins, which were detected in the coffee brew.

### 3.3. Effect of Flaxseed Roasting on Antiradical Capacity (ORAC_FL)

Table 3 shows the effect of flaxseed roasting within the range of selected temperatures on the antiradical capacity of defatted flaxseed meals, measured as scavenging activity against peroxyl radical (ORAC_FL assay).

The roasting process decreased the antiradical capacity of flaxseeds. The antiradical activity of the untreated seeds was significantly greater than that of the seeds roasted even at 160 °C, regardless of the flax cultivar. The lowest values were noted for the seeds roasted at 180 °C and slightly higher for the seeds roasted at 200 °C, but the increase was statistically significant only for the Szafir seeds. This observation is consistent with the results of our previous study [29], which showed that the thermal pre-treatment of flaxseeds (steaming and roasting at various times and temperatures conditions) negatively affected the antiradical activity of aqueous extracts prepared from these seeds. There was a negative linear correlation between the roasting time or temperature and the scavenging activity of aqueous extracts against peroxyl radical. In the present research, there was also a statistically significant negative linear correlation between the roasting temperature and the result of the ORAC_FL assay (r = −0.894, *p* < 0.001).

This finding supports our earlier assumption concerning the formation of MRP products such as melanoidins, which exhibit antiradical activity during roasting at 200 °C. There have been numerous studies [47,51] reporting the antioxidative activity of high MW melanoidin fractions in various food products, both the chelating and radical-scavenging capacity. It was associated with the structural properties of melanoidin molecule as well as the presence of bound-to-melanoidin compounds such as phenolic compounds and low MW Maillard reaction products. The antioxidative capacity of these products may have improved slightly after roasting at 200 °C, as shown in Table 3. However, the antiradical capacity of these seeds was still significantly lower than that of the raw seeds. It showed that the formation of new antioxidative compounds during “dark” roasting was insufficient to overcome the degradation of some native antioxidants in the flaxseed matrix. Further research is necessary to investigate this problem.

### 3.4. Relationship between Protein Profile, MRP, and Antiradical Capacity after Flaxseed Roasting

The relations between the selected variables (the results of linear regression analysis) are shown in Table 4. Principal component analysis (PCA) was applied to show relations between these variables, i.e., the MRP content (both F-MRP and B-MRP), the antiradical capacity (ORAC_FL value) and the percentage of protein fraction at MWs of 13 kDa NR, 19 kDa NR, and 17 kDa R (the protein fractions whose share in the SDS-PAGE NR and R profiles was the most significantly influenced by roasting). PCA helped to derive the factors along which these variables can be classified and to map the tested variants (samples of various flaxseed cultivars roasted at various temperatures) into these factors.

**Figure 3:**
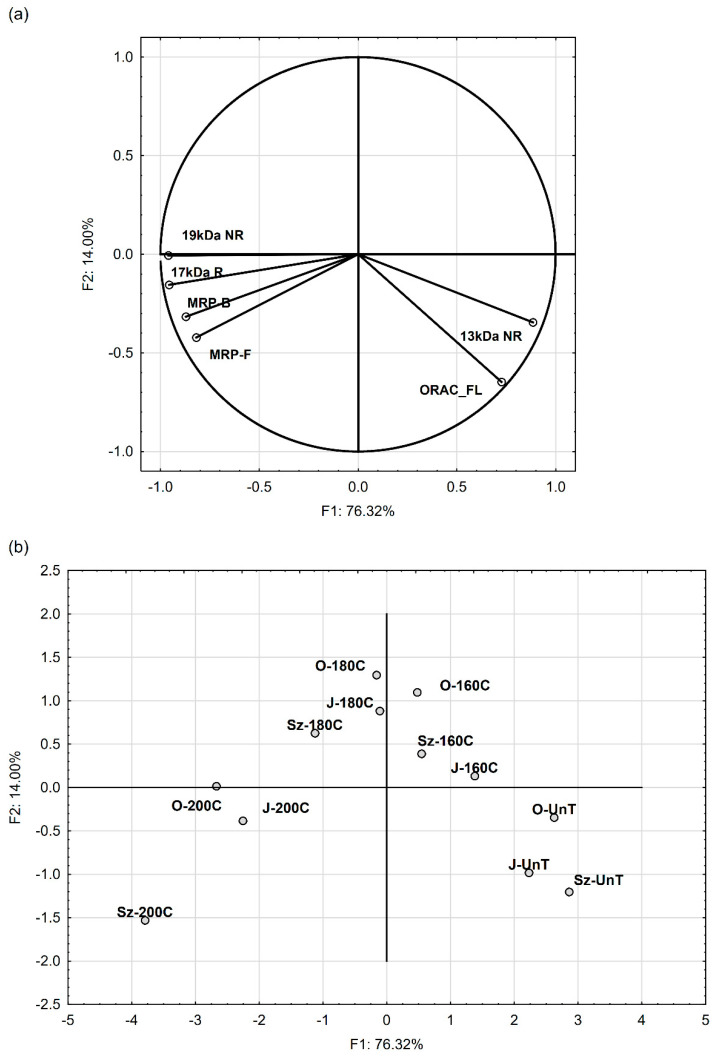
Plots of the first two principal components (F1 and F2): (**a**) variability-factor coordinates plot, (**b**) case-factor coordinates plot; flaxseed cultivars: Oliwin (O), Jantarol (J), and Szafir (Sz); flaxseed treatment: untreated (UnT) and roasted (8 min. at 160 °C, 180 °C and 200 °C).

The analysis showed that the first two components (factor F1 and F2) were the most important elements explaining variation in the data. Because both factors explained approximately 90.3% of the total variance, they were selected for data interpretation (Figure 3) in our study.

The factor coordinates of the variables (Figure 3a) indicate that F1 was mostly explained by the changes in the protein share of 19 kDa NR, 17 kDa R (−0.96), and 13 kDa NR (0.88), and also by the F-MRP and B-MRP content (−0.82 and −0.87, respectively). On the other hand, factor F2 was mostly negatively correlated with the ORAC_FL values (the factor coordinate of this variable was −0.64), but the variable was also positively correlated with factor F1 (0.73).

Factor F1, which explained the total variance of selected variables (76.32%) to the greatest extent, seems to show the significance of the roasting temperature: <180 °C vs. ≥180 °C for the changes observed in the defatted flaxseed meals after roasting. The projection of the variants on the factor-plane F1 × F2 (Figure 3b) shows that the flaxseeds roasted at <180 °C had positive coordinate values for F1, whereas the ones roasted at ≥ 180 °C had negative coordinate values. As far as factor F2 is concerned, this projection shows that the untreated flaxseeds and the ones roasted at 200 °C had negative coordinate values for the factor, whereas the flaxseeds roasted at 160 °C and 180 °C had positive coordinate values, regardless of the flax cultivar.

The regression analysis did not show a significant relation (*p* ≥ 0.05) between the cultivar and any of the selected variables (Table 4). The results suggest that, during flaxseed roasting, these changes occur regardless of the cultivar.

## 4. Conclusions

The most significant changes in the SDS-PAGE profiles of the roasted seeds of each cultivar were observed in the protein fractions at MWs of 13 kDa (decrease) and 19 kDa and 17 kDa (increase). They may have been caused by structural modifications of flaxseed proteins upon thermal treatment as well as oxidation processes and MRP formation. Both F-MRP and B-MRP were formed during the roasting of flaxseeds, regardless of the cultivar. There was a significant relationship between the roasting temperature and B-MRP content. In comparison with the untreated seeds, the roasting process decreased the antiradical capacity of flaxseeds. The MRP formation slightly improved the antioxidative capacity after roasting at 200 °C, but it was still significantly lower than the antioxidative capacity of the raw seeds. It indicated that the MRP formation during “dark” roasting was insufficient to overcome the degradation of some native antioxidants of flaxseeds.

The research provides novel information about key protein fractions that seem to be important changing during heat treatment. However, further characterization of the 13 kDa, 19 kDa and 17 kDa protein bands appears to be important. Our research only shows generally the protein fractions, not identifying specific proteins. In the future, the results of our work should be extended to the identification of specific proteins and peptides (using mass spectrometric analysis, e.g., LC-MS/MS) within the fractions that we have selected. It is also necessary to conduct further research on the contribution of these proteins to the MRP formation mechanism and their possible significance for the antioxidative system of flax seeds. Roasting may also influence the functional properties of flaxseed proteins because the functionality of plant proteins is often determined by their structure. The issues related to the influence of heat treatment on changes in protein profiles of flaxseed and the functionality of flaxseed proteins need to be continued.

## Figures and Tables

**Table 1:** The effect of flaxseed roasting on changes in the protein profile of defatted meals—the SDS-PAGE under non-reducing conditions.

Band	Protein ≈ MW (kDa)	Flaxseed Cultivar	Thermal Treatment			
			Untreated	Roasted		
				160 °C	180 °C	200 °C
Protein contribution (%)						
1a	53	Szafir	10.10 ^a^ ± 0.25	9.65 ^b^ ± 0.13	8.16 ^d^ ± 0.12	8.68 ^c^ ± 0.12
		Oliwin	14.82 ^a^ ± 0.28	13.37 ^b^ ± 0.06	14.25 ^a^ ± 0.38	10.46 ^c^ ± 0.25
		Jantarol	13.62 ^c^ ± 0.03	13.71 ^c^ ± 0.04	15.80 ^a^ ± 0.14	14.39 ^b^ ± 0.13
2a	45	Szafir	13.48 ^a^ ± 0.14	12.40 ^b^ ± 0.05	10.32 ^c^ ± 0.28	7.57 ^d^ ± 0.11
		Oliwin	9.28 ^a^ ± 0.27	4.34 ^b^ ± 0.04	9.10 ^a^ ± 0.13	4.40 ^b^ ± 0.41
		Jantarol	11.80 ^a^ ± 0.55	11.64 ^a^ ± 0.52	11.20 ^a^ ± 0.59	3.29 ^b^ ± 0.37
3a	31	Szafir	8.65 ^d^ ± 0.22	19.29 ^a^ ± 0.10	18.12 ^b^ ± 0.54	17.23 ^c^ ± 0.05
		Oliwin	9.62 ^c^ ± 0.21	18.81 ^a^ ± 0.04	11.85 ^b^ ± 0.04	18.96 ^a^ ± 0.31
		Jantarol	13.48 ^d^ ± 0.22	15.67 ^c^ ± 0.10	17.56 ^b^ ± 0.40	23.29 ^a^ ± 0.50
4a	19	Szafir	15.03 ^d^ ± 0.25	24.94 ^c^ ± 0.34	29.53 ^b^ ± 1.34	41.02 ^a^ ± 0.36
		Oliwin	12.82 ^d^ ± 0.33	25.21 ^c^ ± 0.22	26.70 ^b^ ± 0.11	46.84 ^a^ ± 0.31
		Jantarol	17.31 ^d^ ± 0.09	18.96 ^c^ ± 0.07	27.18 ^b^ ± 0.43	42.31 ^a^ ± 0.54
5a	17	Szafir	3.58 ^a^ ± 0.09	2.13 ^b^ ± 0.04	1.40 ^c^ ± 0.43	0.00 ^d^ ± 0.00
		Oliwin	4.04 ^a^ ± 0.18	2.41 ^b^ ± 0.02	4.13 ^a^ ± 0.20	0.00 ^c^ ± 0.00
		Jantarol	4.67 ^a^ ± 0.00	3.21 ^c^ ± 0.02	4.12 ^b^ ± 0.24	0.00 ^d^ ± 0.00
6a	13	Szafir	25.88 ^a^ ± 0.52	4.61 ^b^ ± 0.16	0.20 ^c^ ± 0.01	0.00 ^c^ ± 0.00
		Oliwin	28.54 ^a^ ± 0.06	10.33 ^b^ ± 0.24	3.74 ^c^ ± 0.13	0.00 ^d^ ± 0.00
		Jantarol	19.06 ^a^ ± 0.09	16.92 ^b^ ± 0.35	2.71 ^c^ ± 0.09	0.00 ^d^ ± 0.00
7a	12	Szafir	4.50 ^c^ ± 0.06	5.04 ^b^ ± 0.11	6.02 ^a^ ± 0.26	3.86 ^d^ ± 0.13
		Oliwin	3.42 ^b^ ± 0.15	3.99 ^b^ ± 0.03	5.62 ^a^ ± 0.02	3.55 ^b^ ± 0.41
		Jantarol	3.48 ^d^ ± 0.02	4.34 ^b^ ± 0.03	4.73 ^a^ ± 0.03	4.06 ^c^ ± 0.04
8a	11	Szafir	8.85 ^c^ ± 0.24	13.21 ^a b^ ± 0.39	12.39 ^b^ ± 0.52	13.57 ^a^ ± 0.18
		Oliwin	7.04 ^a^ ± 0.13	10.38 ^b^ ± 0.01	12.71 ^a^ ± 0.11	10.41 ^b^ ± 0.31
		Jantarol	5.32 ^d^ ± 0.09	6.17 ^c^ ± 0.04	7.31 ^b^ ± 0.17	8.00 ^a^ ± 0.09
Proteins at MW range						
Ia	>55	Szafir	1.54 ^a^ ± 0.02	1.29 ^b^ ± 0.01	0.00 ^c^ ± 0.00	0.00 ^c^ ± 0.00
		Oliwin	2.44 ^a^ ± 0.05	2.19 ^b^ ± 0.13	0.00 ^c^ ± 0.00	0.00 ^c^ ± 0.00
		Jantarol	2.07 ^a^ ± 0.01	1.32 ^b^ ± 0.13	0.00 ^c^ ± 0.00	0.00 ^c^ ± 0.00
IIa	54–16	Szafir	59.03 ^d^ ± 0.00	75.86 ^c^ ± 0.33	81.41 ^b^ ± 0.78	82.58 ^a^ ± 0.06
		Oliwin	58.57 ^d^ ± 0.10	73.13 ^c^ ± 0.34	77.93 ^b^ ± 0.22	86.06 ^a^ ± 0.10
		Jantarol	70.07 ^d^ ± 0.01	71.27 ^c^ ± 0.40	85.26 ^b^ ± 0.11	87.94 ^a^ ± 0.14
IIIa	15–10	Szafir	39.45 ^a^ ± 0.00	22.86 ^b^ ± 0.34	18.61 ^c^ ± 0.79	17.42 ^d^ ± 0.06
		Oliwin	39.00 ^a^ ± 0.08	24.69 ^b^ ± 0.21	22.07 ^c^ ± 0.22	13.96 ^d^ ± 0.10
		Jantarol	27.86 ^a^ ± 0.02	27.42 ^b^ ± 0.29	14.74 ^c^ ± 0.11	12.06 ^d^ ± 0.14

Protein contribution (%)—the percentage share of protein in the total protein content. MW—protein molecular weight. The means ± SD marked with different superscript letters in a row are significantly different (one-way ANOVA, *p* < 0.05, and post hoc Tukey’s test).

**Table 2:** The effect of flaxseed roasting on changes in the protein profile of defatted meals—the SDS-PAGE under reducing conditions.

Band	Protein ≈ MW (kDa)	Flaxseed Cultivar	Thermal Treatment			
			Untreated	Roasted		
				160 °C	180 °C	200 °C
Protein contribution (%)						
1b	32	Szafir	21.59 ^a^ ± 0.52	17.90 ^b^ ± 0.80	15.71 ^c^ ± 0.67	6.88 ^d^ ± 0.27
		Oliwin	30.82 ^a^ ± 1.17	27.13 ^b^ ± 0.63	24.47 ^c^ ± 0.13	20.08 ^d^ ± 0.77
		Jantarol	26.31 ^a^ ± 1.24	26.65 ^a^ ± 0.12	25.61 ^a^ ± 0.04	20.96 ^b^ ± 0.01
2b	27	Szafir	9.15 ^b^ ± 0.54	8.86 ^b^ ± 0.24	10.71 ^a^ ± 0.29	4.99 ^c^ ± 0.14
		Oliwin	10.15 ^c^ ± 1.43	11.59 ^b,c^ ± 0.48	13.09 ^a,b^ ± 0.24	14.19 ^a^ ± 0.93
		Jantarol	11.61 ^d^ ± 1.30	13.15 ^b^ ± 0.01	15.01 ^a^ ± 0.07	15.73 ^a^ ± 0.10
3b	20	Szafir	9.99 ^a^ ± 0.11	5.60 ^c^ ± 0.12	6.90 ^b^ ± 0.11	0.00 ^d^ ± 0.00
		Oliwin	8.39 ^a^ ± 0.02	7.12 ^b^ ± 0.15	6.78 ^c^ ± 0.01	4.71 ^d^ ± 0.05
		Jantarol	7.57 ^b^ ± 0.04	8.20 ^a^ ± 0.05	7.42 ^c^ ± 0.04	0.00 ^d^ ± 0.00
4b	17	Szafir	20.04 ^c^ ± 0.02	20.80 ^c^ ± 0.53	41.33 ^b^ ± 0.58	63.76 ^a^ ± 0.04
		Oliwin	22.14 ^d^ ± 0.04	28.70 ^c^ ± 0.08	37.85 ^b^ ± 0.00	52.24 ^a^ ± 0.02
		Jantarol	23.07 ^d^ ± 0.13	27.68 ^c^ ± 0.10	33.60 ^b^ ± 0.32	49.48 ^a^ ± 0.25
5b	15	Szafir	2.76 ^a^ ± 0.00	1.81 ^b^ ± 0.04	1.37 ^c^ ± 0.01	0.00 ^d^ ± 0.00
		Oliwin	2.69 ^a^ ± 0.06	0.00 ^b^ ± 0.00	0.00 ^b^ ± 0.00	0.00 ^b^ ± 0.00
		Jantarol	3.37 ^a^ ± 0.07	1.02 ^b^ ± 0.00	0.00 ^c^ ± 0.00	0.00 ^c^ ± 0.00
6b	11	Szafir	20.60 ^b^ ± 0.35	24.96 ^a^ ± 0.06	10.61 ^d^ ± 0.38	11.8 ^c^ ± 0.08
		Oliwin	10.38 ^b^ ± 0.07	13.96 ^a^ ± 0.41	8.54 ^c^ ± 0.07	5.58 ^d^ ± 0.06
		Jantarol	11.56 ^a^ ± 0.02	11.77 ^a^ ± 0.35	8.78 ^b^ ± 0.08	4.45 ^c^ ± 0.04
Proteins at MW range						
Ib	>36	Szafir	4.99 ^b^ ± 0.03	3.29 ^d^ ± 0.06	6.49 ^a^ ± 0.21	4.61 ^c^ ± 0.03
		Oliwin	6.84 ^a^ ± 0.06	4.81 ^c^ ± 0.05	5.91 ^b^ ± 0.14	2.63 ^d^ ± 0.03
		Jantarol	6.63 ^a^ ± 0.01	3.69 ^c^ ± 0.01	5.01 ^b^ ± 0.06	6.11 ^a^ ± 0.06
IIb	35–17	Szafir	63.15 ^c^ ± 0.12	53.36 ^d^ ± 1.20	75.23 ^b^ ± 0.08	83.51 ^a^ ± 0.12
		Oliwin	71.68 ^d^ ± 0.38	75.28 ^c^ ± 0.07	82.75 ^b^ ± 0.14	91.79 ^a^ ± 0.09
		Jantarol	75.33 ^d^ ± 0.04	78.37 ^c^ ± 0.21	82.27 ^b^ ± 0.31	89.45 ^a^ ± 0.12
IIIb	16–10	Szafir	29.39 ^b^ ± 0.08	39.33 ^a^ ± 0.22	14.40 ^c^ ± 0.42	11.81 ^d^ ± 0.08
		Oliwin	21.01 ^a^ ± 0.30	18.26 ^b^ ± 0.03	11.35 ^c^ ± 0.01	5.58 ^d^ ± 0.06
		Jantarol	16.96 ^a^ ± 0.05	17.13 ^a^ ± 0.23	12.73 ^b^ ± 0.25	4.45 ^c^ ± 0.04

Explanations as in Table 1.

**Table 3:** The antiradical capacity of the untreated and roasted flaxseeds—ORAC_FL assay.

Flaxseed Cultivar	Thermal Treatment				*p*
	Untreated	Roasted			
		160 °C	180 °C	200 °C	
Szafir	136.05 ± 0.87 ^d,C^	85.99 ± 1.81 ^c,B^	57.94 ± 2.98 ^a,A^	70.17 ± 2.06 ^b,B^	<0.0001
Oliwin	100.82 ± 1.73 ^c,A^	59.75 ± 5.14 ^a,A^	56.92 ± 2.15 ^a,A^	62.03 ± 2.52 ^a,A^	<0.0001
Jantarol	130.70 ± 4.23 ^c,B^	88.26 ± 3.65 ^b,B^	69.74 ± 7.30 ^a,B^	71.95 ± 5.84 ^a,B^	<0.0001
*p*	<0.0001	<0.0001	0.0069	0.0121	

The results were expressed as Trolox equivalent μM/g flaxseed meal. The means ± SD marked with different superscript letters are significantly different (one-way ANOVA, *p* < 0.05, and post hoc Tukey’s test): a, b, c, d in a row—pre-treatment effect; A, B, C in a column—cultivar effect.

**Table 4:** The relations between the selected variables, the roasting conditions and cultivar.

Variabilities	Correlation Coefficient r					
	17 kDa R	13 kDa NR	19 kDa NR	ORAC_FL	MRP-F	MRP-B
13 kDa NR	−0.746 **	-	-	-	-	-
19 kDa NR	0.905 ***	−0.853 ***	-	-	-	-
ORAC_FL	−0.618 *	0.806 **	−0.657 *	-	-	-
MRP-F	0.829 **	−0.608 *	0.745 **	−0.343 NS	-	-
MRP-B	0.879 ***	−0.632 *	0.837 **	−0.437 NS	0.731 **	-
Roasting	0.694 *	−0.914 ***	0.781 **	−0.894 ***	0.497 NS	0.584 *
Cultivar	−0.091 NS	0.080 NS	−0.046 NS	0.041 NS	−0.235 NS	−0.235 NS

Simple linear regression equation y = Ax + B (the linear least squares method), *** *p* < 0.001, ** *p* < 0.01, * *p* < 0.05, NS—the finding is not statistically significant (*p* ≥ 0.05).

## Supplementary Materials

The following are available online at https://www.mdpi.com/2304-8158/9/10/1383/s1, Table S1: Composition [g kg^−1^] of untreated and thermally pre-treated flaxseeds.
