# Supplementary material for: The Effect of Roasting on the Protein Profile and Antiradical Capacity of Flaxseed Meal
Source: Foods. 2020 Sep 30;9(10):1383. doi: 10.3390/foods9101383 (PMC7599758; doi:10.3390/foods9101383)
Supplement: Supplementary file 1 [file foods-09-01383-s001.pdf]

Supplementary materials

Article: Waszkowiak, K., Mikołajczak, B. The Effect of Roasting on the Protein Profile and Antiradical Capacity of Flaxseed Meal. *Foods* **2020**

Table S1 Composition [g kg^{-1}] of untreated and thermally pre-treated flaxseeds

Cultivar	Composition	Untreated	Roasted		
			160 °C, 8 min	180 °C, 8 min	200 °C, 8 min
Szafir	Moisture	69.7±0.3^{b}	43.3±3.9^{a}	42.7±5.3^{a}	42.8±5.3^{a}
	Protein (N x 6.25)	220.9±8.7^{a}	217.8±4.0^{a}	216.4±1.6^{a}	216.4±3.3^{a}
	Fat	386.4±0.7^{a}	426.3±4.5^{b}	430.2±2.4^{b}	420.8±4.7^{b}
Oliwin	Moisture	58.2±8.7^{b}	42.1±1.6^{a}	46.3±3.5^{a}	41.1±0.3^{a}
	Protein (N x 6.25)	213.7±0.7^{a}	222.9±4.0^{b}	223.9±2.6^{b}	230.1±2.1^{b}
	Fat	400.1±1.1^{a}	435.7±3.8^{b}	435.2±3.2^{b}	444.0±9.9^{b}
Jantarol	Moisture	54.2±1.3^{b}	46.6±1.1^{a}	46.5±1.6^{a}	42.5±0.4^{a}
	Protein (N x 6.25)	170.4±3.7^{a}	174.4±3.8^{a}	176.5±2.6^{a}	176.8±7.3^{a}
	Fat	421.7±6.0^{a}	459.4±0.9^{b}	463.9±6.4^{b}	459.3±8.8^{b}

The means±SD marked with different superscript letters (a, b) in a row are significantly different (one-way ANOVA, $P<0.05$, and post hoc Tukey’s test).
